# Molecular epidemiology of multidrug-resistant clinical isolates of *Acinetobacter baumannii*: A 10-year analysis in a large tertiary care university hospital in central Europe with international admissions

**DOI:** 10.1007/s00508-017-1242-7

**Published:** 2017-08-03

**Authors:** Luigi Segagni Lusignani, Peter Starzengruber, Verena Dosch, Ojan Assadian, Elisabeth Presterl, Magda Diab-Elschahawi

**Affiliations:** Department of Infection Control and Hospital Epidemiology, Medical University of Vienna, Waehringer Guertel 18–20, 1090 Vienna, Austria

**Keywords:** Acinetobacter baumannii, Multi-drug resistance, Nosocomial infections, Molecular typing, Intensive care unit

## Abstract

**Background:**

Over the last 10 years, multidrug resistant *Acinetobacter baumannii *has been spreading worldwide as emerging microorganisms that negatively impact on the outcome of in-hospital patients.

**Methods:**

Between 2007 and 2016, all isolates of patients of the Vienna General Hospital (VGH), tested positive for multidrug resistant *Acinetobacter baumannii *(MDR *A. baumannii*) strains, were investigated with respect to their genetic relationship. Patient medical histories were reviewed in order to collect discriminating factors related to MDR *A. baumannii* colonization or infection.

**Results:**

A total of 79 isolates of 76 patients were obtained. For 44 of them (55.7%) the first diagnosis ward was an intensive care unit (ICU). A total of 10 genotype clusters were identified and 35 cases (44.3%) of in-hospital acquisition in our institution could be detected. Multidrug resistant *Acinetobacter baumannii* isolates were acquired before admission to our hospital in 44 cases (55.7%) and in 31 (70.5%) they belonged to patients who had previous exposure to the healthcare setting of high prevalence countries for MDR *A. baumannii*.

**Conclusion:**

Patients admitted to our hospital with a previous healthcare contact in a high prevalence country for multidrug resistant *Acinetobacter baumannii* should be screened before admission to high-risk wards. Isolation of these patients until microbiological results could reduce negative outcome in these wards.

## Introduction

*Acinetobacter baumannii *is an emerging opportunistic microorganism [1, 2] which may cause severe nosocomial infections [3–13]. International spread of multidrug-resistant* A. baumannii* (MDR *A. baumannii*) has been described worldwide and international transfer of colonized patients has led to the introduction and subsequent epidemic spread of MDR *A. baumannii* strains from southern to northern European countries [14, 15].

Exposure to healthcare systems of countries with high-prevalence (HPCs) of multidrug-resistant microorganisms (MDRO) has been described as a risk factor for the spread and acquisition of these microorganisms [16–18]. Although Austria is situated in the heart of the European Union with historic and current strong ties to East and Southeastern European countries, so far no epidemiologic investigation has been conducted to analyses possible international transmission of MDR *A. baumannii *in this geographic region. Austria hosts some 1600000 individuals (18% of the population) from East European member states, who regularly visit their home countries [19]. More than one third of these guests live in Vienna. The Vienna General Hospital (VGH), a large university teaching hospital, serves as specialist tertiary care center for severely ill patients from all parts of Austria as well as for patients from surrounding countries frequently admitted for burns or transplantation surgery.

The national antimicrobial resistance rate in Austria is low compared to the European average where in 2015 the overall percentage of invasive *A. baumannii* strains resistant to carbapenem was 6.4% [20]. From 2014 to 2016 the mean number of *A. baumannii *isolated at VGH was 130 per year. The yearly rates of MDRO were 25%. Beginning in 2007, the VGH registered an alarming increase of *A. baumannii *strains resistant to at least four antibiotic classes (penicillins, cephalosporins, fluoroquinolones and carbapenems), particularly in patients admitted to ICUs.

The aim of this study was to characterize all MDR *A. baumannii *strains collected between June 2007 and May 2016 at the VGH and to describe epidemiological patterns of the colonized and infected patients.

## Methods

### Setting

The VGH is a 1922 bed tertiary care university hospital. It is the reference hospital for both Austria and Central Eastern Europe. The facility provides 149 beds in 21 ICUs and 93 beds in 12 intermediate care units (IMCs) for treating critically ill patients. From 2007 through 2015 the number of patients admitted to the VGH increased by 6%, reaching 105,000 admissions in 2015. Patients admitted to ICUs are not routinely screened for MDRO including MDR *A. baumannii*.

### Data collection and processing

During the 10-year study period, all routinely collected microbiological results for MDR *A. baumannii* were prospectively monitored by the Department of Infection Control and Hospital Epidemiology until patients’ discharge date. Patients’ contacts with previous healthcare facilities abroad were retrospectively collected with electronic sources and, if not available, obtained by information from the ward physician. Contact was defined as any exposure to a healthcare system in the last 4 weeks before admission to the VGH, independent of the kind of healthcare setting, time spent there and reason of admission to the VGH. Outpatients clinic as well as hospital admissions were included.

The European Centre for Disease Prevention and Control (ECDC) antimicrobial resistance statistical data report from 2007 through 2015 were yearly used to classify MDR *A. baumannii *high-prevalence EU countries [21, 22]; data for countries outside of EU were extrapolated from current country literature [1, 23, 24].

### Case definition and microbiological identification

The MDR *A. baumannii* was defined as any isolate resistant to penicillins, cephalosporins, fluoroquinolones and carpabenems. The MDR *A. baumannii* strains were identified by using cultures on Columbia agar containing 5% sheep blood (Becton Dickinson, Heidelberg, Germany) and on in-house manufactured MacConkey agar plates with crystal violet. The first isolate of each patient was identified to the species level using matrix-assisted laser desorption/ionization time-of-flight mass spectrometry (MALDI-TOF MS). Antibiotic susceptibility testing was performed using the disk-diffusion method (Mast Diagnostica, Reinfeld, Germany) on an in-house manufactured Mueller-Hinton agar. It was carried out according to the European Committee on Antimicrobial Susceptibility Testing (EUCAST) methods. The minimum inhibitory concentration (MIC) was identified by the E‑test method (bioMérieux, Marcy l-Etoile, France). Molecular genotyping was performed by means of an automated repetitive-sequence-based PCR (rep-PCR) assay on the DIVERSILAB® system (DL, bioMérieux). The US Center for Diseases Prevention and Control criteria [25, 26] were used to discern VGH origin of infection/colonization. When *A. baumannii* was isolated 48 h after VGH patient hospitalization, infection or colonization were considered as healthcare acquired. If a microbiology test was undertaken at patient admission and was positive within 48 h since hospitalization, *A. baumannii *was considered acquired before hospital admission and if the patient was admitted or transferred to the VGH with a known positive MDR *A. baumannii* result.

### Statistical analysis

Data analysis was performed using SPSS Statistics 23.0 (IBM, Armonk, NY). Categorical data were analyzed using the *χ* ^2^-test and *p*-value <0.05 was considered significant.

## Results

A total of 76 patients with microbiological positive test for MDR *A. baumannii* were recorded and of these 44 (56%) were isolated from skin and wounds, 24 (30%) from respiratory tract specimens, 5 (6%) from rectal swabs, 4 (5%) from central venous catheters and 2 (3%) from abscess aspirates of the patients. A total of 47 (62%) colonized and 29 (38%) infected patients were admitted to altogether 41 wards (13 medical wards, 8 surgical wards, 16 ICUs and 4 IMCs). Of the 76 patients, 50 (66%) were male and 26 (34%) female, 7 were children under 8 years old (6 male, 1 female) and the age of the overall patients ranged between 0 days and 78 years (mean 43.3 years, SD ± 21 years). The duration of the hospital stay at the VGH lasted between 7 and 186 days (mean 58 days, SD ± 42 days). All patients spent at least part of their hospital stay in ICUs (range: 4–186 days, mean stay 32 days, SD ± 33 days) and additionally 7 of them in an IMC (range: 2–162 days, mean stay 13 days, SD ± 16 days). Multiple trauma was the cause for the hospital stay in 29% (22/79) of patients, followed by acute lung diseases (20%; 15/76), burns (12%; 9/76), and cancer (9%; 7/76). Of the patients 15 died during the hospital stay and 8 (53%) thereof due to pneumonia and sepsis. By reviewing the clinical charts of these patients, a causal relationship between MDR* A. baumannii* infection and death was found in 6 cases.

The results of susceptibility testing showed that out of 79 *A. baumannii* isolates resistant to meropenem, 21 (27%) were additionally resistant to imipenem and 45 of the 79 isolates (62%) were moreover resistant to aminoglycosides. No isolate showed resistance to colistin. A significant trend of increasing incidence of MDR over the 9‑year period was found (Cochran-Armitage *p* < 0.05).

All 79 isolates were genotyped by the Diversilab® system. By using a similarity threshold of 95%, the analysis of the data identified 23 different genotypes, of which 13 were only observed once and showed a Dice similarity coefficient to all other strains between 81% and 92%. The 10 remaining genotypes were found repeatedly and they could be grouped into 10 clusters (I–X), consisting of a minimum of 2 to a maximum of 21 strains (Table 1). In cluster VI two strains belonged to the same person and differed in antibiotic susceptibility. Cluster VII consisted of two strains belonging to the same person but differed in antibiotic resistance. One patient had at the same time one strain belonging to cluster X and one strain observed only once, those showed different antibiotic susceptibility. All other strains of the 10 clusters belonged to different patients.

**Table 1:** Multidrug resistance *Acinetobacter baumannii *genotypes identified and extended period in the Vienna University Hospital

Genotype	Period	Duration^a^	Isolates (*n*)
*Cluster*			
I	08/2007–07/2009	48	18
II	02/2008–02/2009	13	6
III	12/2008–04/2015	79	21
IV	10/2009–03/2010	6	3
V	12/2010–09/2013	34	2
VI	01/2011–10/2015	58	3
VII	02/2011–05/2011	4	2
VIII	05/2013–05/2015	25	5
IX	01/2014–03/2016	24	4
X	12/2014–02/2015	3	2
Total	08/2007–03/2016	294	66
Single genotype			13
Total			79

^a^in months

While with clusters II, IV, VII and X there was no overlap in the times of stay of all patients, with cluster III there was a close occurrence in the times of stay (3 months). With clusters V (28 months), VI (39 months), VIII (16 months) and IX (18 months) the time gaps up between the times of stay of the patients was far more high.

**Fig. 1:**
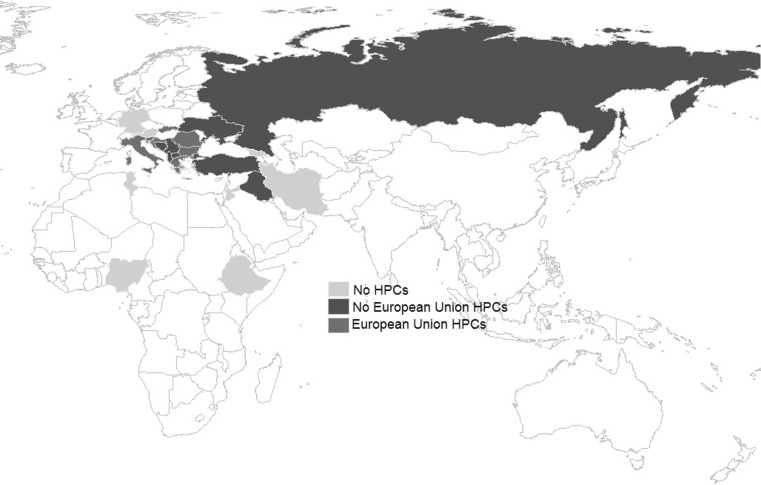
Country of origin of the patients and country distribution according to MDR *A. baumannii* prevalence

Of the MDR *A. baumannii* strains 56% (44/79) were first isolated in ICUs, 28% (22/79) in surgical departments, 15% (12/79) in medical and 1 in an IMC unit. Of the MDR *A. baumannii* strains 56% (44/79) were acquired before patient admission to the VGH and 44% (35/79) during their hospitalization. Regarding the nosocomial acquired *A. baumannii* strains, 6 index patients could be identified as source of secondary transmission. Within cluster I, this occurred between 2 patients after a 3 weeks time frame; within cluster II the time frame of acquisition between 2 patients was 10 days. Both transmissions occurred in ICUs. Of the 6 acquired *A. baumannii* documented within cluster III, the mean time frame was 93.2 days (±SD 89 days) and 71.4% (5/6) of them occurred in ICUs. Within cluster VI, *A. baumannii* time frame of acquisition between index patient and secondary case was 240 days, 11 days within cluster VIII and 8 days within cluster IX. All acquisitions within these three clusters occurred in ICUs. Of the 79 patients 31 (41%) had no contact with healthcare systems of HPCs for MDR *A. baumannii* in the 4 weeks before VGH admission, 77% (24/31) of them were Austrian citizens with no foreign background or travel history. Of the 76 patients 27 (36%) had a previous contact with a healthcare setting of an EU-HPC for MDR *A. baumannii* and 67% (18/27) of them with Rumanian hospitals. Within the patients that had previous contact with a healthcare setting of HPCs outside the EU (18/76; 24%), 13 (72%) were transferred from hospitals of the former Yugoslavia (Table 2). The country of origin of the patients and country distribution according to MDR *A. baumannii *prevalence are illustrated in Fig. 1.

**Table 2:** Overview of patient last contact with healthcare systems before admission to Vienna General Hospital (VGH) and indication of whether multidrug-resistant *Acinetobacter baumannii* (MDR *A.* *baumannii*) was acquired before or after VGH admission

Country of healthcare last contact^a^	Total number of patients	MDR *A. baumannii* acquired before admission	MDR *A. baumannii* acquired after admission
*European Union HPCs* ^*b*^			
Romania	18	15	3
Croatia	4	3	1
Bulgaria	1	1	0
Greece	1	0	1
Italy	1	0	1
Slovakia	1	1	0
Slovenia	1	1	0
*Total*	*27*	*21*	*6*
*Non-European Union HPCs* ^*b*^			
Bosnia and Herzegovina	5	3	2
Serbia and Montenegro	5	0	5
Macedonia	2	2	0
Kosovo	1	0	1
Albania	1	1	0
Russia	1	0	1
Ukraine	1	0	1
Turkey	1	0	1
Iraq	1	1	0
*Total*	*18*	*7*	*11*
*No HPCs* ^*b*^			
Austria	24	9	15
Germany	1	1	0
Georgia	1	0	1
Jordan	1	1	0
Iran	1	0	1
Tunisia	1	1	0
Nigeria	1	0	1
Ethiopia	1	1	0
*Total*	*31*	*13*	*18*

^a^within the past 4 weeks

^**b**^high prevalence countries for multidrug-resistant *Acinetobacter baumannii*

**Fig. 2:**
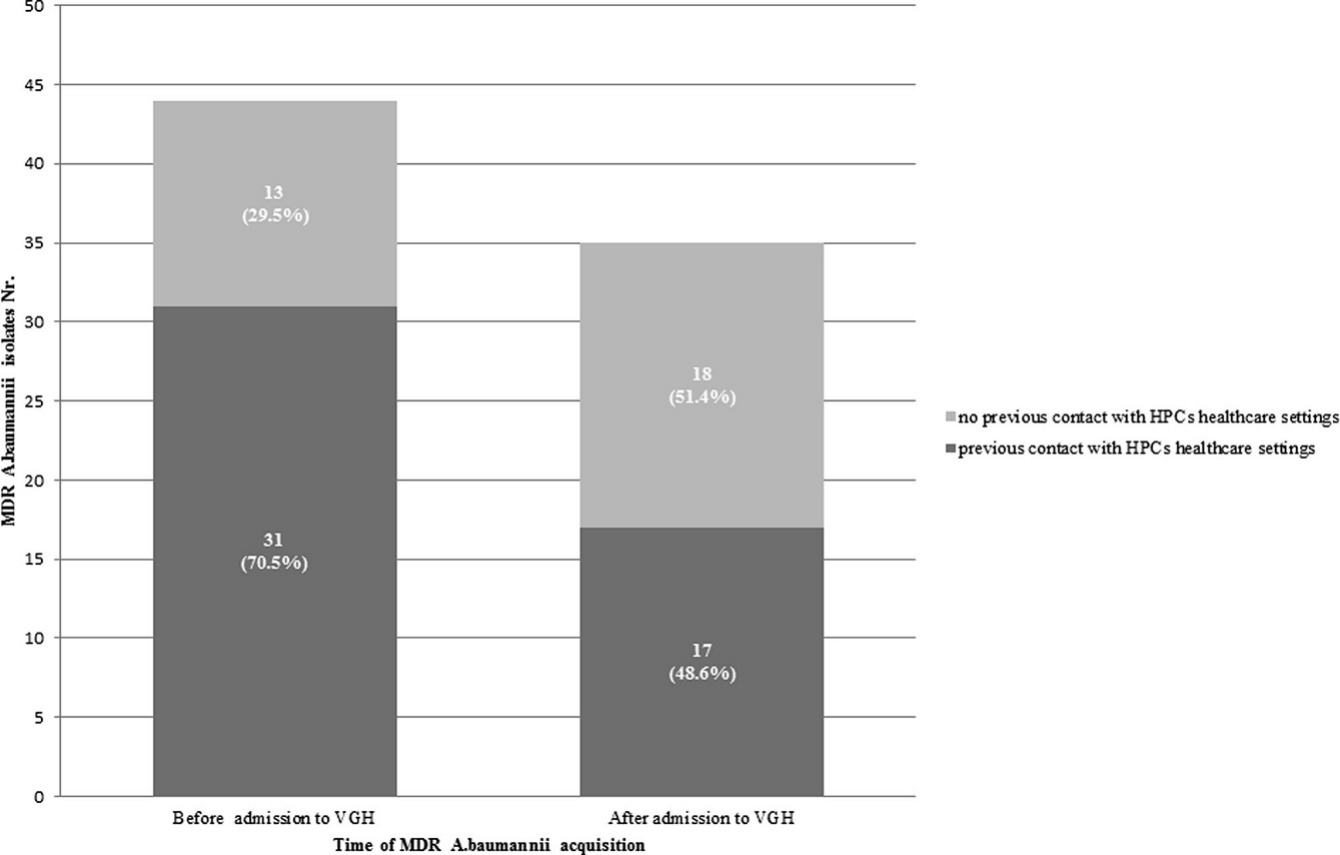
Strains of multidrug resistance *Acinetobacter baumannii* identified in 2007–2016, patients’ previous contact with healthcare systems of high prevalence countries (HPCs) for multidrug-resistant *Acinetobacter baumannii* and time of *Acinetobacter baumannii* acquisition in Vienna General Hospital (VGH)

Within the patients that acquired *A. baumannii* strains before admission to the VGH, previous contact with healthcare settings of HPCs for MDR *A. baumannii* was found in 71% (31/44). Within the patients that acquired *A. baumannii* after VGH admission this was 49% (17/35, χ^2^-test 3.9; *p* < 0.05) (Fig. 2).

Of 6 identified index cases belonging to the genotyped clusters, where *A. baumannii* was nosocomially acquired (I, II, III, IV, VI, VIII, IX), 5 (83.3%) had a previous contact to a healthcare system of a HPC for MDR *A. baumannii*: 2 from an EU country (Romania) and 3 from a country outside the EU (Serbia and Macedonia).

## Discussion

Our results show that ICUs are the most frequent setting for MDR *A. baumannii* in-hospital acquisition. Both colonized and infected patients were severely ill and spent all or part of their hospital stay in an ICU. Almost half of these cases were polytrauma patients or had suffered severe burns. The ICUs were also the setting were more than half of the patients belonging to the identified genotyped clusters that were cared for. These findings support the role of indwelling devices and invasive procedures for MDR *A. baumannii* colonization or infection [27].

Interestingly we found a long time frame between strain acquisitions in three genotyped clusters (VI, VIII and IX). We decided, however, not to split these clusters as the excessive time of MDR *A. baumannii* acquisition within these clusters may be due to the persistence of *A. baumannii* in a hospital environment and confirms the importance of contamination of hospital inanimate surfaces in the transmission of these pathogens [28]. This could be, under certain conditions, far longer than the 5 months reported in literature. Nevertheless, cases of *A. baumannii* colistin resistance have been increasingly reported in Europe [29]; however, no such isolate was found in our hospital in the last 9 years, which reflects the drug resistant microorganism rates in our country [20].

Because of the worldwide alarming trend in carpabenem and multidrug resistance, specific efforts of our epidemiological research was focused on the previous contact of the MDR *A. baumannii* patients with healthcare systems in other countries. Approximately two thirds (48/76) of the patients were exposed to a healthcare setting of HPCs for MDR *A. baumannii* in the previous 4 weeks before VGH hospitalization and the same proportion (31/44) was observed within those who acquired *A. baumannii* strains before admission in our hospital. Furthermore 5 out of 6 identified index patients were admitted to healthcare services of HPCs for MDR *A. baumannii*, confirming exposure to these healthcare settings as a major risk for *A. baumannii* colonization or infection.

We chose not to differentiate between type of healthcare exposure and length of hospitalization in such countries because no evidence was found in the literature to be a relevant determinant of MDRO acquisition [17]. Even if past studies used longer exposure periods to a healthcare system abroad [16, 29], we chose a 4-week time frame before admission to our hospital, in order to avoid possible errors in the collection of the patient hstory records.

Our study had some limitations. Firstly, we did not examine other recognized factors associated with MDR *A. baumannii* colonization and infection, such as the antibiotic use before hospitalization in our hospital. Then, as a systematic microbiological screening of fellow patients was not conducted during the observation period, the role of asymptomatically colonized patients in the transmission chain remained unknown. In his research Joly-Guillou [5] indicated a ratio of colonized patients to infected patients of 10:1 for *Acinetobacter* in a clinical unit and concluded that the patients with symptoms of *Acinetobacter* infection are likely to represent the tip of the colonization iceberg and significant undetected dissemination is also likely to be occurring. This suggests that in our case the colonized patients could have been far more than the 47 identified in our hospital. The strengths of our study were the long-time observation period and that not only invasive but also non-invasive isolates of *A. baumannii* were investigated. As far as we know, no other study has collected data on the molecular characterization of MDR *A. baumannii* strains of colonized and infected patients for such a long period of time.

In conclusion, the findings of our study warrant the implementation of a targeted risk-adjusted screening policy for *A. baumannii* for all patients before admission to ICUs. Given the high percentage of MDR *A. baumannii* cases that were imported from foreign countries in our study population, we advise in accordance with the recent recommendations of the European Centre for Diseases Prevention and Control regarding the screening regimen for carbapenem-resistant *A. baumanni *[30], to screen all patients, transferred from foreign countries with high prevalence rates MDR *A. baumannii*.
